# The “what, why, and how?” of story completion in health services research: a scoping review

**DOI:** 10.1186/s12874-024-02274-7

**Published:** 2024-07-23

**Authors:** Candelyn Yu Pong, Nicola J. Roberts, Elaine Lum

**Affiliations:** 1 Health Services & Systems Research, Duke-NUS Medical School, National University of Singapore, Singapore, Singapore; 2 Centre for Population Health Research & Implementation, SingHealth Duke-NUS Academic Medical Centre, Singapore, Singapore; 3 School of Clinical Sciences, Faculty of Health, Queensland University of Technology, Brisbane, Australia; 4 School of Health and Social Care, Edinburgh Napier University, Edinburgh, UK

**Keywords:** Story completion, Health services research, Study design, Qualitative research

## Abstract

**Background:**

The story completion method provides a different way of doing qualitative research. We note the emergent popularity of this method in health-related research, while much remains to be negotiated in terms of best practices for such studies. This scoping review aims to provide a synthesis on how researchers have used the story completion method in health services research. We offer implications for research and practice for further discussion by the scholarly community.

**Methods:**

We used the JBI methodology for scoping reviews. Six databases were searched for published literature till March 1, 2023: Medline, Embase, CINAHL, PsycINFO, SAGE Journals Online databases, and SAGE Research Methods. We included primary studies of any study design using the story completion method in health services research.

**Results:**

A total of 17 studies were included. Findings suggest that the story completion method is useful for research on sensitive topics, and affords the use of comparative study designs and large sample sizes which may be difficult with conventional qualitative research methods. More than 80% of included studies used story completion as the sole method. However, the data collected from this method were limited in terms of the inferences that can be drawn; and richness of participant responses may vary widely. Less than 30% of included studies reported piloting of the story stems. Most studies were conducted online and analyzed qualitatively, though the story stem design and sample size varied widely.

**Conclusion:**

The story completion method, with its attendant affordances for larger sample sizes, comparative study designs, and streamlined data collection is an innovative and useful stand-alone or adjunct qualitative method for health services research.

**Supplementary Information:**

The online version contains supplementary material available at 10.1186/s12874-024-02274-7.

## Background

Qualitative methods increasingly underpin robust population health research, health services research, and implementation research [1–3]. The insights provided by qualitative methods allow us to appropriately design, execute, and evaluate a plethora of healthcare programs and innovations, including digital health and AI-augmented healthcare [4–6].

Qualitative methods used in these fields include interviews and focus groups. These methods are often time and resource intensive [7], and arguably less efficacious in eliciting uncensored views especially for topics that are socio-culturally sensitive [8]. In that, participants may adjust their positions on an issue to align with what they perceive as accepted social or cultural discourses, perhaps to avoid potential repercussions [9].

Story completion is a method not often used in population health, health services, and implementation research. Given its attributes, apart from being used on its own to explore socio-culturally sensitive topics, story completion promises to be a useful adjunct to semi-structured interviews and focus groups. The story completion method has already garnered much interest in the scholarly community, with several published discussions regarding its utility and issues [10, 11], flexibility as a method across disciplines [12], and potential for decolonizing research methodologies [13].

### What is story completion?

Story completion, first used in quantitative developmental psychology research and in psychoanalysis as a projective technique for clinical assessment, was subsequently re-developed as a qualitative method by Kitzinger for feminist research [8]. Importantly, Kitzinger re-conceptualized story completion in the mid-1990s as a way “to access not just psychological meanings but also social discourses” [8]. More recently, Clarke and colleagues re-ignited interest in this method with the publication of a special issue “Using Story Completion Methods in Qualitative Research” in the Qualitative Research in Psychology journal [8]. Although story completion originated as a pen-and-paper task, this method has been increasingly administered online. Hence, the moniker, digital story completion.

In typical story completion studies, participants are presented with one or several hypothetical scenarios that act as writing prompts (story stems) and asked to complete the story however they like (Table 1). In example 1, researchers used story completion as a stand-alone qualitative method to collect narratives from Australian adults regarding their views on the COVID-19 restrictions implemented, and how it affected their health and well-being [14]. The story completion method was chosen due to its ability to examine social discourses, meanings, norms, and assumptions; and researchers were interested to understand how individuals would react to constantly changing situations, such as COVID-19 restrictions [14]. In example 2, researchers also used story completion as a stand-alone method to explore how evangelical Christians perceive depression [15]. Story completion method was chosen in this case due to the stigma associated with depression or mental health in general; and as this method does not explicitly obtain respondents’ personal experiences or views, it reduces the risk of social desirability bias [15].

**Table 1:**
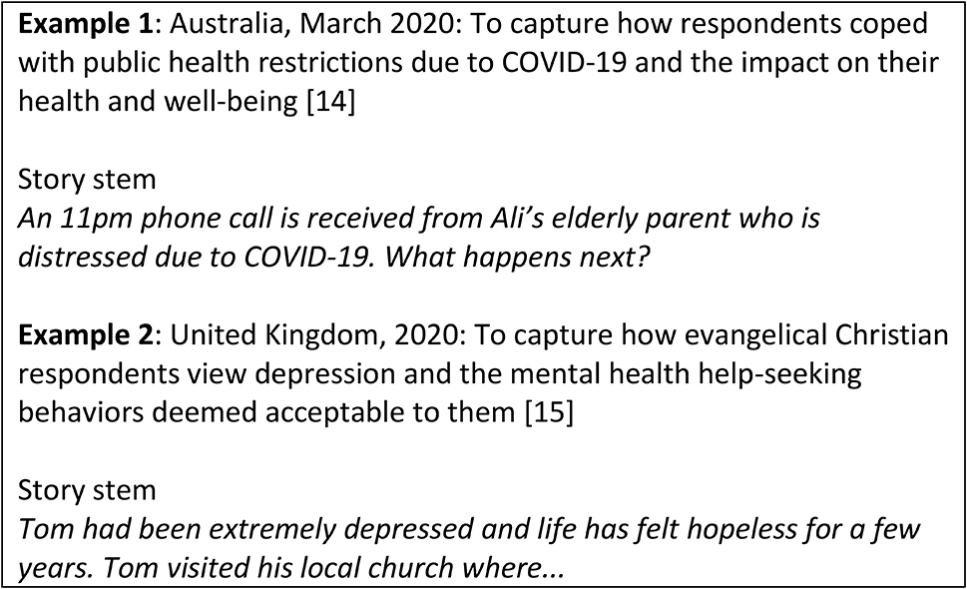
Examples of story completion task

A key advantage of the story completion method is its ability to side-step solely direct personal experiences to include socio-cultural discourse and representations, which enables researchers to understand meaning-making frameworks of a particular social group [10]. Other notable advantages of the method include the ability to accommodate larger samples of participants relative to traditional qualitative methods, and the contentious use of comparative study designs, uncommon in qualitative research [10].

A perceived weakness of this method has to do with the invitation to participants to be imaginative when responding to the story stem, triggering some researchers (and users of research) to be concerned that “anything goes.” To alleviate this concern, we recognize the bi-directional connection between imagination and experience, where imagination is influenced by an individual’s experiences [16] and “experiences are partly constituted through the stories within [one’s] socio-cultural landscapes [17]. So, despite its apparent playfulness, story completion holds merit as a sole method and as a useful adjunct to traditional qualitative methods in multiple- or mixed-methods studies.

### Rationale for this review

We observed a steady increase via PubMed in the number of studies using story completion for health-related research in the last five years. While each study justifies and explains its use, it is our opinion that much needs to be clarified and negotiated about best practices for this method. For example, how should story stems be derived? How and when to use comparator groups? How large should the sample be to yield adequate data for meaningful analysis? These questions pertain to future discussions about best practices or ‘shoulds’. However, we first need to understand the current landscape. In this scoping review we elicited how the story completion method has been used in health-related research. Specifically, we were interested in: (a) the target populations and/or health conditions, (b) the study designs used, (c) how story stems were derived, (d) how data were analyzed, (e) other research methods used to triangulate data from the story completion method, and (f) strengths and weaknesses of the method stated by study authors. Our findings serve as a useful resource or starting point for health services researchers interested in using the story completion method, when planning or designing their study.

## Methods

### Search strategy

This study was carried out in accordance with the JBI methodology for scoping review [18]. The protocol was published on Open Science Framework (available here: https://osf.io/rk2e6/) [19]. We developed a search strategy using the PRESS guidelines [20] and consulted university librarians for refinement around the following key terms: story completion and health services research (Additional File 1). We searched six databases: Medline, Embase, CINAHL, PsycINFO, SAGE Journals Online databases, and SAGE Research Methods for published literature till March 1, 2023.

### Eligibility criteria

Inclusion criteria: a primary study of any study design using the story completion method in health services research. For the purposes of this review, we defined story completion as a type of qualitative research method where study participants are asked to complete a story based on an assigned story “stem” or opening [8], and health services research as an interdisciplinary study of scientific investigation that explores how social determinants, financial policies, organizational systems and structures, medical technology, and individual actions influence cost, access, quality of healthcare, and also our well-being and health [21]. This definition of health services research does not confine it to the provision of health services or health structures, but also includes the exploration of how social determinants and individual behaviors affect health and well-being. The World Health Organization recognizes social determinants such as social exclusion and discrimination as important factors that can affect access to healthcare and health equity in negative ways [22]. Hence, studies investigating perceptions of potentially stigmatizing conditions or sexual orientations which are likely to influence how/whether those individuals seek help/healthcare have been included. Studies were excluded if they were editorials, commentaries, discussion papers, methodological papers (non-empirical), conference papers, systematic reviews, meta-analyses, or study protocols.

### Selection of studies

Three researchers (CP, NJR, EL) independently conducted title/abstract and full text screening of studies captured by the search strategy. Conflicts at both screening stages were resolved through discussion by two lead researchers (NJR, EL). Covidence^®^, a web-based software for conducting reviews (Veritas Health Innovation, Melbourne, Australia) and Endnote 20 (Clarivate Analytics, PA, USA) were used for screening and managing citations respectively. Studies in languages other than English were translated using ChatGPT (OpenAI, CA, USA) and screened by two researchers (CP, EL), to determine eligibility.

### Data extraction and data analysis

A standardized form was developed for data extraction using Google Forms. The following data were extracted: publication year, author, country of study, characteristics of the study population, study aim(s), study design, description of the story completion study, sample size, how story stems were derived, how data was captured and analyzed including type of analysis (e.g. Braun & Clarke’s reflexive thematic analysis, etc.), other research methods used to triangulate data (e.g. semi-structured interviews, surveys, focus groups, etc.), reported strengths and weaknesses of the story completion method, assumptions and underlying theories.

The form was piloted by three researchers (CP, NJR, EL) using three included studies, and refined accordingly. How we operationalized data extraction is shown in Additional File 2. Two researchers (CP, EL) independently completed data extraction for the remaining studies. Publication year and sample size were extracted as numerical values. Other data points expressed as textual data were summarized rather than extracted verbatim from included studies, apart from author, country of study, and study aims. For example, data point “characteristics of the study population” were summarized as “Australia-based adults aged 18 and above during the COVID-19 pandemic”, “adolescents aged 14–25 years old with complex regional pain syndrome” and so forth. Descriptive statistics, where appropriate, were used to summarize extracted data in Excel^®^ (Version 1808 (Microsoft)). For example, to provide a numerical count of how many included studies were single country versus multi-country, and so forth.

## Results

**Fig. 1:**
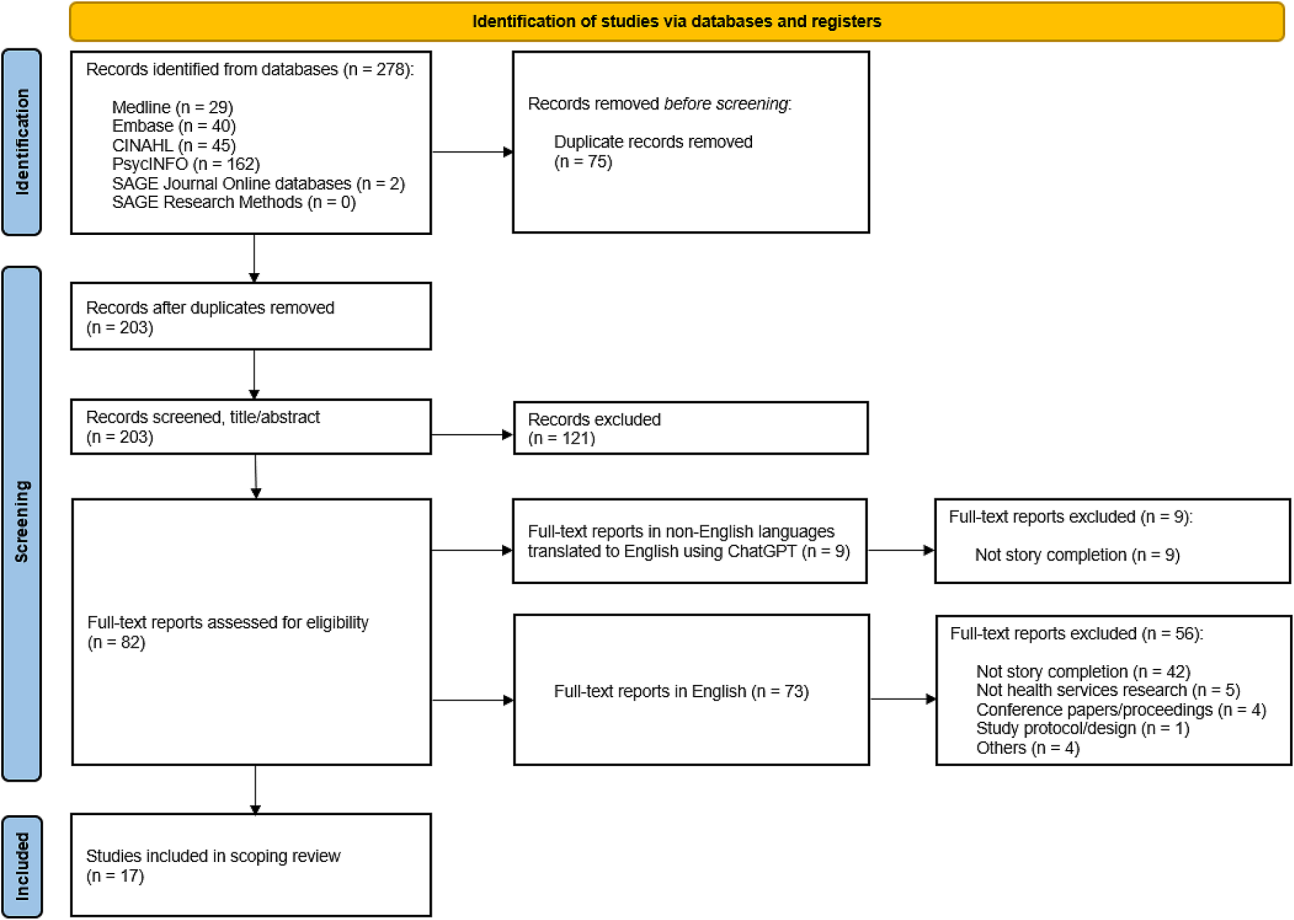
PRISMA-scoping review flow diagram [18]

The search yielded 278 studies. After removing 75 duplicates, 203 studies remained for screening. At full text screening stage there were nine studies reported in languages other than English which were translated using ChatGPT; these did not meet eligibility criteria and were excluded. A total of 17 studies were included in this review (Fig. 1). The list of included studies is provided as Additional File 3.

### Study characteristics

The main characteristics of included studies are summarized in Table 2. The majority were single country studies (15/17, 88·2%), originating from the European region (9/17, 52·9%) and Western Pacific region (4/17, 23·5%). Most of the studies were published between 2021 and 2023 (9/17, 52·9%). In terms of the study design used, of the 17 studies, 13 (76·5%) were qualitative, three (3/17, 17·6%) used a mixed-method design, and one (1/17, 5·9%) used a multi-method design (Table 2). We used the following definitions for mixed-method and multi-method studies, respectively. Mixed-method studies use two or more methods in a single research project comprising both qualitative and quantitative approaches, that involves the connection, integration, or linking of these two approaches [23]. Multi-method studies use two or more solely qualitative or solely quantitative methods in a single research project [24]. In addition, although the story completion method allows for comparative study designs, this was adopted by only three studies (3/17, 17·6%) [25–27].

**Table 2:** Study characteristics

Study characteristics	Number of papers, *n* = 17 (%)^a^
**Country of study** ^b^	
Single country study	15 (88.2)
- European Region	9 (52.9)
- Western Pacific Region	4 (23.5)
- Region of the Americas	2 (11.8)
Multi country study^c^	2 (11.8)
**Publication year**	
2000 and before	1 (5.9)
2001 to 2010	2 (11.8)
2011 to 2020	5 (29.4)
2021 to 2023	9 (52.9)
**Study design**	
Qualitative study	13 (76.5)
Mixed-method study^d^	3 (17.6)
Multi-method study^e^	1 (5.9)
**Type of data analysis**	
Qualitative analysis	14 (82.4)
- Braun and Clarke’s reflexive thematic analysis	12 (70.6)
- Jackson and Mazzei’s “thinking with theory” approach	1 (5.9)
- Wetherell, Taylor, and Yates’ discourse analysis	1 (5.9)
Quantitative analysis^f^	3 (17.6)
- Statistical analysis^g^	2 (11.8)
- Text analysis and statistical analysis^g, h^	1 (5.9)
**Assumptions and underlying theories**	
Not reported	7 (41.2)
Epistemology: Constructionist	4 (23.5)
Critical realism	2 (11.8)
Epistemology: Contextualist	2 (11.8)
Feminist post-structuralist	1 (5.9)
More-than-human theory and post qualitative inquiry	1 (5.9)

^a^Percentages may not add up to 100% due to rounding

^b^Countries are categorized based on World Health Organization’s country groupings [28]

^c^Two studies were conducted in multiple regions; one was conducted in the African region, European region, region of the Americas, South-East Asia region, and Western Pacific region, while another was conducted in the European region, region of the Americas, and Western Pacific region

^d^Mixed-method study: The use of two or more methods in a single research project comprising both qualitative and quantitative approaches, that involves the connection, integration, or linking of these two approaches [23]

^e^Multi-method study: The use of two or more solely qualitative or solely quantitative methods in a single research project [24]

^f^Quantitative analysis: Three studies used a quantitative approach to analyze story completion data

^g^Statistical analysis include descriptive statistics such as mean, standard deviation as well as inferential statistics such as t-test, ANOVA, and ANCOVA

^h^Text analysis in this case refers to the Linguistic Inquiry and Word Count software [29]

### Description of the story completion study

Most studies asked participants to complete one story stem each, with the exception of five studies (5/17, 29·4%) which asked each participant to complete either two [30–32] or three story stems [33, 34]. Of these five studies, four provided multiple story stems to allow a diversity of illnesses, genders, socio-economic groups, or occupations to be included in the stem [30, 32–34]; while one did not provide a rationale for having multiple stems. Story stems provided were fairly brief, consisting of two to five short sentences. Examples of story stems can be found in Table 1.

For single story stem studies (*n* = 12), most provided the same story opening to every participant, except three (3/12, 25.0%) which adopted a comparative design where study authors developed two story stems of the same narrative but with different protagonists in terms of gender [25, 26] or occupation [27]. Participants were allocated [25] or randomly allocated to either stem [26, 27]. Another study (1/12, 8.3%) randomized each participant to one of three story stems pertaining to the research, with results from each stem analyzed separately [35]. Of the five multi-story stems studies, three (3/5, 60.0%) provided the story openings in the same order for participants [31, 33, 34]. Two studies (2/5, 40.0%) counterbalanced the order of the story openings with half the participants presented with the first story stem followed by the second story stem while the other half were presented with the second story stem followed by the first [30, 32].

Most studies provided participant guidelines for either time (minutes) and/or length (number of words/ characters/ sentences) for story responses (10/17, 58·8%), though these varied widely among studies (Table 3). Sample size varied widely among studies as well; ranging from 17 to as large as 227 (Table 3).

**Table 3:** Overview of included studies

Study no.	Paper	Study aim(s)	Population studied	Target health condition	Time and length guidelines for story responses	Sample size
1	Diniz 2020	To explore the association between nurses’ perception of patients’ socio-economic status and the dehumanizing inferences made pertaining to their pain	Female nurses	Chronic disease: Chronic pain	N.A.	50
2	Hayfield 2022	To explore young adults’ perception of peri/menopause	Psychology undergraduates	Peri/menopause	Length: Minimally 200 words long	102
3	Jones 2020	To explore how young people with complex regional pain syndrome view their future	Adolescents aged 14–25	Chronic disease: Complex regional pain syndrome	Length: Minimally 900 characters	50
4	Jones 2009	To explore the construct of future temporal horizon using two indicators: future time perspective which includes a story completion task to estimate how far into the future an individual normally plans, and delay discounting; as well as to understand the influence of two variables, smoking and gender, that may modulate temporal horizon	Adults (age range not specified)	Smoking	N.A.	227
5	Lloyd 2022a	To explore evangelical Christians’ perception(s) of mental health help-seeking behaviors	Evangelical Christians aged 18 and above	Mental health: Depression	Time: Minimally 10 min Length: Approximately 10 lines or 200 words	110
6	Lloyd 2022b	To explore evangelical Christians’ perception(s) of self-harm	Evangelical Christians	Mental health: Self-harm	Time: Minimally 10 min Length: Approximately 200 words or 10 lines	101
7	Lupton 2021	To explore the role of digital technologies on individuals’ health seeking behaviors	Adults aged 18 and above	No target health condition; study is about health seeking behaviors	Length: Minimally four sentences	43
8	Moller 2019	To explore young people’s perception(s) of fatness, and its association with gender and professional competence	Adolescents aged 15–24	No target health condition; study is about people’s perception of fatness	Time: Minimally 10 min	203
9	Nimbley 2021	To explore the differences in social, affective and cognitive language used by young people with complex regional pain syndrome relative to their peers who are pain-free	Adolescents aged 14–25	Chronic disease: Complex regional pain syndrome	Length: Minimally 10 lines long, with a minimum of 900 characters	97
10	Olstein 2021	To explore gay men’s perception(s) of suicidal thoughts among people in their community, and if supporting their fellow gay friends would be seen as protective	Gay men aged 18 and above	Mental health in general	Time: Minimally 10 min	23
11	Scholz 2020	To explore the differences in opinions between health professionals and consumer representatives working in the healthcare industry on the decision-making processes regarding mental health	Consumer representatives working in the healthcare industry and mental health professionals	Mental health in general	N.A.	34
12	Scott 2022	To explore the perceptions of Australian adults on societal norms for parents, more specifically that of parental food provision	Adults aged 18 and above	No target health condition; study is about social norms surrounding family food provision	Time: Maximum 10–20 min Length: Minimally 2–3 sentences	75
13	Shah-Beckley 2021	To explore psychology undergraduates and therapists’ perception(s) of heterosexual refusal	Therapists and psychology undergraduates	No target health condition; study is about heterosexual refusal	N.A.	71
14	Tichenor 1977	To explore physical therapy students and therapists’ perception(s) of cancer	Physical therapists and physical therapy students	Cancer	Time: Maximum of one hour to formulate responses, and 10 min for the actual writeup	17
15	Tischner 2019	To explore young people’s perception(s) of health, appearance, and weight loss	Young adults aged 18–24	Weight loss	N.A.	170
16	Vaughan 2022	To explore how Australians coped with the COVID-19 restrictions, more specifically the impact of these restrictions on individuals’ well-being and mental health	Adults aged 18 and above	Mental health: COVID-19	N.A.	52
17	Walsh 2010	To explore young adults’ perception(s) of anorexia and bulimia	Young adults aged 18–23	Mental health: Anorexia and bulimia	N.A.	22

### Populations studied and health conditions

Study aims, populations studied, and health conditions are shown in Table 3. Study participants were recruited from general populations or subgroups of general populations (e.g. adolescents, gay men), or were working adults in the healthcare industry and/or students in a health-related course. Most studies targeted a particular health condition (13/17, 76·5%). Of those that did, six studies focused on mental health conditions (6/17, 35·3%), three on chronic diseases (3/17, 17·6%), and one on cancer (1/17, 5·9%).

Study aims of some included studies are socio-culturally sensitive. For example, in the study by Lloyd et al 2022 one of the aims was to “explore how self-harm is perceived” [36], while Walsh et al 2010 aimed to “explore the ways in which ‘anorexic’ and ‘bulimic’ young women are discursively constructed by those who neither self-identify as ‘eating disordered’ nor are involved in ‘eating disorder’ interventions” [32].

### How story stems were derived

Story stems were constructed by study authors in most studies, except for four studies (4/17, 23.5%) where study authors reported using either published literature or a theoretical framework to inform the development of story stems [27, 33, 36, 37], and one study (1/17, 5.9%) which derived and modified the story stems based on the Wallace (1956) measure [31, 38]. The Wallace measure estimates how far into the future a person typically plans (future time perspective) and consists of two types of questions concerning timeframes about future actions or outcomes [38]. For example, the first type of question may ask participants to list 10 events that will occur in their lives and the age they would expect to be for each event. The second type of question asks participants to write endings to story stems (i.e. story completion) and to indicate the duration in which the story occurred (e.g. “x” minutes, days, years).

The majority of studies did not pre-test the story stems (12/17, 70·6%); of those that did, they were either piloted to ensure clarity [15, 30, 35, 36] or to prevent potential narrowing of responses [9]. Most studies adopted third-person story stem(s), except two studies (2/17, 11.8%) that used a first-person story stem to allow participants to reflect on their perceived future [39, 40].

### How data was captured and analyzed

Twelve out of 17 studies (70·6%) administered the story completion task online. The remaining five studies (5/17, 29.4%) were administered either in-person [31, 34], a combination of both in-person and online [9], or did not report the mode of data collection [30, 32]. Most studies analyzed the data qualitatively (14/17, 82·4%), with Braun & Clarke’s reflexive thematic analysis [41] as the most commonly used approach (12/17, 70·6%) (Table 2). However, three studies (3/17, 17.6%) applied a quantitative approach to the analysis of story completion data, as follows. The study by Nimbley et al. 2021 analyzed the stories collected using the Linguistic Inquiry and Word Count (LIWC) program which identified and coded words against pre-selected categories pertaining to positive or negative emotions, social, and cognitive dimensions determined by study authors [39]. The LIWC program subsequently generated quantitative data in the form of frequencies (counts) and proportions of words against these categories, which were further analyzed using statistical programs such as SPSS [39]. In the study by Jones et al. 2009, participants were asked to complete two story stems and to also indicate the duration in which the story occurred (e.g. minutes, days, years). The duration was quantitatively analyzed (salient to their research question), while the stories collected were not subjected to further analysis [31]. The study by Tichenor et al. 1977 analyzed the stories collected via deductive coding using a schema of 12 categories pre-developed by study authors, then assigning a frequency score [34]. The rates of expression for each of these categories were standardized through dividing the frequency scores by the number of words written by participants and multiplying this number by a constant of 1000 [34].

### Research methods used to triangulate data

The majority of studies did not use other research methods to triangulate the data from the story completion method, with the exception of two (2/17, 11.8%) that used surveys [30, 34] and one that used semi-structured interviews (1/17, 5·9%) [40]. Of the two studies that used surveys to triangulate data, one conducted the survey prior to the story completion task [30] whilst the other did not specify the order in which the tasks were carried out [34]. The sole study that used semi-structured interviews conducted them after the story completion task to explore the stories crafted by participants in greater detail [40].

### Reported strengths and weaknesses of the story completion method

The story completion method is reported to be useful for exploring sensitive topics and vulnerable populations [9, 15, 25, 32, 36, 37, 42] as it does not require participants to reveal their personal experiences [14, 27, 33]. Instead of actual behaviors, story completion method uncovers participants’ unconscious and subconscious patterns and ways of sense-making as well as perceptions towards a given scenario, beyond their lived experiences [9, 14, 15, 27, 35, 42], thereby reducing the risk of social desirability bias [15, 36].

Hence, this method reportedly allows study authors the potential to obtain rich data pertaining to both individual and collective experiences of major social events and problems [14, 30] that may not be elicited through more conventional data collection methods [26, 27]. Additionally, data from a larger group of participants can be collected more efficiently, relative to other forms of qualitative methods [26, 40].

However, the story completion method is not without weaknesses. Study authors discerned that as the story completion method does not explicitly obtain participants’ personal experiences [15, 27, 36], it limits the inferences that can be derived from the findings [14, 37]. When crafting responses, participants may exaggerate the protagonist’s life to produce a “good” story that they would otherwise not have done in a more conventional data collection method [32] or orientate their responses to include more social elements than what they would have otherwise given due to the type of story stem provided [40].

Additionally, study authors reported that participants’ engagement with the story stem varied widely. Some would provide complex and detailed responses while others produced superficial and short stories [25], and some may misinterpret the task and provide a theoretical account of the assigned story opening instead of completing the story [25, 26]. Study authors also noted that in common with other qualitative research, it is hard to recruit male participants [26].

### Underpinning philosophy

Most studies specified the ontology, epistemology, or theoretical lens used (10/17, 58·8%). The top three were social constructionism (4/17, 23·5%), critical realism (2/17, 11·8%), and epistemic contextualism (2/17, 11·8%) (Table 2). Study authors deemed the story completion method to be compatible with their selected underpinning philosophy, which was in turn used to inform interpretation of the narratives collected.

## Discussion

This scoping review provides a synthesis of how the story completion method has been used in health services research thus far. Our findings serve as a useful resource for health services researchers interested in exploring and using the story completion method, when planning or designing their study. We found several distinct advantages of the story completion method, suggesting its usefulness as either a sole or adjunct approach to undertaking qualitative research, provided its shortcomings are mitigated.

First, the story completion method enables large sample sizes as the collection of data can be done in a relatively efficient way, compared to traditional qualitative methods such as semi-structured interviews. Several studies included in this scoping review reported sample sizes of over 100 [9, 15, 26, 31, 36, 37], the largest being 227 [31]. In contrast, the average sample size was between 18 and 45 in a recent systematic analysis of sample sizes for interview-based studies published over a 15-year period in health research journals [43]. Second, the story completion method can accommodate comparative study designs, which is unusual in qualitative methods, and useful for systematically eliciting differences in variables salient to the research question (e.g. male/female, novice/expert, and so forth). An overview of the story completion method by Clarke et al. 2019 underscores that this advantage allows a more “nuanced understanding of how a particular phenomenon is socially constructed” [8]. Third, story completion is especially appropriate for research on sensitive topics as it minimizes the risk of social desirability bias, a common problem reported in qualitative health research literature [44]. This is because in contrast to qualitative methods such as semi-structured interviews, the story completion method allows participants to respond to socio-culturally sensitive topics as a third party and to participate anonymously (assuming the story stem references a third party e.g. “Tom”, “Ali”, and data collection was conducted via an online platform). In our review, conditions that carry social stigma such as mental health issues and eating disorders, were target health conditions among included studies.

The shortcomings of the story completion method reported by study authors are acknowledged in methodological discussions about this innovative approach [10, 11]. The non-intrusive data collection afforded by the story completion method might limit the inferences study authors can draw from the findings as responses might not reflect participants’ lived experiences [14, 15, 27, 36, 37]. However, Clarke et al. 2019 cautions that whether this constitutes a problem depends on the ontological stance taken: “Essentialist/realist/(post)positivist researchers may be concerned that data may not reflect or predict “real-life” behaviour. By contrast, for social constructionist or critical realist researchers interested in the sociocultural meanings or discourses people draw on when writing their stories, this critique holds no water” [8].

Additionally, story completion is a fixed self-administered task unlike other qualitative research methods such as semi-structured interviews or focus groups where researchers and participants interact to co-shape the research-in-progress. Hence, some participants may misinterpret the task [25, 26] or provide responses that fall short of the study authors’ requirements or expectations. When confronted with such data, researchers need to judge whether these responses are sufficiently meaningful to warrant inclusion in the dataset for analysis [11]. Misinterpretation of the story completion task can be mitigated by piloting to ensure clarity [15, 30, 35, 36] or to prevent potential narrowing of responses [9]. Yet, piloting of story stems was conducted by less than a third of included studies.

We note that most studies used story completion as the sole method rather than as an adjunct method. For example, other quantitative or qualitative research methods were not used to triangulate data from the story completion method. Admittedly, some research questions may not require more than a single method. However, the wider literature recognizes the potentially complementary pairing of the story completion method with another method such as semi-structured interviews [11, 12].

Most studies adopted a qualitative approach to data analysis, with Braun & Clarke’s reflexive thematic analysis [41] being the most prominently used. Story stems were brief to allow participants the freedom to construct their own stories; and story stem design varied widely, with the most common being participants completing a single third-person story stem, with the same story opening provided to all.

Since the COVID-19 pandemic, the pace of research has sped up significantly [45]. Researchers engaged in population health, health services, or implementation research have worked on ways to accelerate actionable outputs without compromising scientific rigor; for example, rapid qualitative analysis to reduce the time taken to analyze qualitative data [46, 47] and methods to hasten on-the-ground implementation [48]. The story completion method, with its attendant affordances for larger sample sizes, comparative study designs, and streamlined data collection adds to these innovative methods.

### Limitations and strengths

There are some limitations to this review. First, we may have missed capturing some studies as the search was restricted to peer-reviewed articles and we did not manually search the reference lists of included papers to identify potential studies for inclusion. Second, we did not include an assessment of the reporting quality of included studies. We attempted to assess the reporting quality of 14 out of 17 included studies which conducted qualitative analysis on collected data, using an established checklist for reporting qualitative research — the Consolidated Criteria for Reporting Qualitative Research, COREQ [49]. However, many items on the COREQ checklist were neither appropriate nor relevant to the reporting of story completion studies; for example, interview guide, repeat interviews, field notes, participant checking, and so forth. We are mindful of salient scholarly critique regarding COREQ’s trustworthiness and reliability in reflecting the quality of reporting [50]. Therefore, we could not justifiably adapt COREQ for the purposes of this study.

Strengths of this review include having an extensive search strategy and broad inclusion criteria, allowing us to retrieve as many relevant studies as possible. University librarians were consulted for refinement of search strategy and included studies were not limited to a particular search period or geographical area. We piloted our data extraction form to evaluate its ability to capture relevant study information. Issues were flagged and the form was revised accordingly prior to actual extraction by two researchers.

### Implications for research and practice

Currently, there are no universally agreed best practice nor reporting standard for the story completion method in health services research. Given the various ways in which the story completion method has been used in this scoping review, we offer several suggestions for research and practice for further discussions by the scholarly community.

First, piloting is crucial and recommended by key proponents of the story completion method [8, 11]. Story completion is a fixed task unlike other qualitative methods such as semi-structured interviews or focus groups where it is possible to iteratively modify the questions as participants co-shape the research-in-progress. Piloting is a smart way to ensure that both the instructions and story stem(s) provided to participants are clear, to prevent misinterpretations.

Second, consider using other quantitative or qualitative research methods to triangulate or corroborate the data from the story completion method if thorough investigation of the research question(s) requires more than one method. While a methodological strength of the story completion method is that it uncovers participants’ patterns and ways of sense-making beyond their lived experiences [9, 14, 15, 27, 35, 42], this also means that there may be limited inferences that can be drawn from the findings since responses may not reflect participants’ realities. Hence, pairing story completion with a story-mediated interview, for example, may lend further insights [12].

Third, as story completion studies are markedly different from traditional qualitative research methods, having an agreed set of reporting criteria for such studies will be useful for health services researchers. For example, a minimalist set of reporting criteria could comprise the 10-item JBI critical appraisal checklist for qualitative research [51] plus a description of the study design, development of the story stems, number of participants/sample size, and how participants completed the task in terms of modality, the number of story stems per participant, and sequence of story stem presentation.

## Conclusion

The story completion method is an exciting and innovative way of doing qualitative research, and has the potential to be used more widely. This scoping review generated a comprehensive summary of how the story completion method has been used in health-related research. Findings and suggestions for research and practice serve as useful resources for researchers interested in experimenting with and adopting the story completion method in their work.

### Electronic supplementary material

Below is the link to the electronic supplementary material.

Supplementary Material 1

Supplementary Material 2

Supplementary Material 3

Supplementary Material 4

## Data Availability

The dataset supporting the conclusions of this article are available in the Open Science Framework repository, https://osf.io/rk2e6/.
