# Supplementary material for: The “what, why, and how?” of story completion in health services research: a scoping review
Source: BMC Med Res Methodol. 2024 Jul 23;24:159. doi: 10.1186/s12874-024-02274-7 (PMC11265486; doi:10.1186/s12874-024-02274-7)
Supplement: Supplementary file 1 — Supplementary Material 1 [file 12874_2024_2274_MOESM1_ESM.pdf]

Additional file 1. Search strategies

Medline (Ovid)

Search conducted on March 1, 2023

Query	Search string	Results retrieved
1	"story complet*".tw.	126
2	health.sh.	25033
3	"health*".af.	7069899
4	"health care".af.	1064530
5	"health service*".af.	630257
6	2 or 3 or 4 or 5	7069899
7	1 and 6	29

Embase

Search conducted on March 1, 2023

Query	Search string	Results retrieved
#1	'story complet*':ab,ti	157
#2	'health'/exp	861,810
#3	health*	10,025,921
#4	'health care'	2,076,645
#5	'health service*'	873,805
#6	#2 OR #3 OR #4 OR #5	10,082,476
#7	#1 AND #6	40

CINAHL

Search conducted on March 1, 2023

Query	Search string	Results retrieved
S1	TI "story complet*" OR AB "story complet*"	82
S2	(MH "Health+")	445,403
S3	TX health*	4,911,131
S4	TX "health care"	1,030,293
S5	TX "health service*"	783,696
S6	S2 OR S3 OR S4 OR S5	4,923,108
S7	S1 AND S6	45

PsycINFO

Search conducted on March 1, 2023

Query	Search string	Results retrieved
1	"story complet*".ab,ti.	393
2	health.mh.	1438
3	"health*".af.	2332936
4	"health care".af.	507932
5	"health service*".af.	326480
6	2 or 3 or 4 or 5	2332936
7	1 and 6	162

SAGE Journal Online databases

Search conducted on March 1, 2023

Query	Search string	Results retrieved
1	(Abstract "story complet*") OR (Title "story complet*")	2

SAGE Research Methods

Search conducted on March 1, 2023

Query	Search string	Results retrieved
1	Title: 'story complet*' OR Abstract: 'story complet*'	0
