# Supplementary material for: The “what, why, and how?” of story completion in health services research: a scoping review
Source: BMC Med Res Methodol. 2024 Jul 23;24:159. doi: 10.1186/s12874-024-02274-7 (PMC11265486; doi:10.1186/s12874-024-02274-7)
Supplement: Supplementary file 2 — Supplementary Material 2 [file 12874_2024_2274_MOESM2_ESM.pdf]

Additional File 2. Data extraction

Data point	Description of data extraction	Data type
Publication year	Publication year of included study	Numerical
Authors	Last name of first author	Text
Country of study	Specify all countries where the study was conducted. When no country is specifically stated, specify the country of the first author	Text
Characteristics of the study population	Specify the population and/or health condition that is being studied	Summary text
Study aim(s)	Specify aim(s) of the study	Text
Study design	Specify the study design, e.g. mixed-method, multi-method, qualitative	Summary text (as papers may not explicitly state study design)
Description of the story completion study	Provide a short description of the story completion study, including how many stories to be completed per participant, how many versions of the story stem were used, instructions to participants, sequence — before or after an interview (if any), any other information related to this data point	Summary text
Sample size	Sample size of the story completion study	Numerical
How story stems were derived	Specify who constructed the story stem, whether developed based on a framework/theory, whether the story stem was pre-tested, as well as the story stem used	Summary text
How data was captured and analyzed including type of analysis	Specify whether the data was collected online or offline, the platform used to collect data, and describe the type of analysis used (e.g. Braun & Clarke's reflexive thematic analysis)	Summary text
Research methods used to triangulate data	Specify the research methods used to triangulate data from story completion component, if any	Summary text
Reported strengths and weaknesses of the story completion method	List the strengths and weaknesses of the story completion method as stated in the paper	Summary text
Assumptions and underlying theories	Describe the assumptions and underlying theories as stated in the paper	Summary text
