# Supplementary material for: The “what, why, and how?” of story completion in health services research: a scoping review
Source: BMC Med Res Methodol. 2024 Jul 23;24:159. doi: 10.1186/s12874-024-02274-7 (PMC11265486; doi:10.1186/s12874-024-02274-7)
Supplement: Supplementary file 3 — Supplementary Material 3 [file 12874_2024_2274_MOESM3_ESM.pdf]

Additional file 3. List of studies in scoping review

List of included studies (n=17)

1. Diniz E, Castro P, Bousfield A, Figueira Bernardes S. Classism and dehumanization in chronic pain: A qualitative study of nurses' inferences about women of different socio-economic status. British journal of health psychology. 2020;25(1):152-70.
2. Hayfield N, Campbell C. Students' representations of menopause and perimenopause: Out of control bodies and empathetic expert doctors. Sex Roles: A Journal of Research. 2022:No-Specified.
3. Jones A, Caes L, Eccleston C, Noel M, Rugg T, Jordan A. Loss-adjusting: Young People's Constructions of a Future Living With Complex Regional Pain Syndrome. The Clinical journal of pain. 2020;36(12):932-9.
4. Jones BA, Landes RD, Yi R, Bickel WK. Temporal horizon: Modulation by smoking status and gender. Drug and Alcohol Dependence. 2009;104(Suppl1):S87-S93.
5. Lloyd CEM, Mengistu BS, Reid G. "His Main Problem Was Not Being in a Relationship With God": Perceptions of Depression, Help-Seeking, and Treatment in Evangelical Christianity. Frontiers in psychology. 2022;13:831534.
6. Lloyd CEM, Panagopoulos MC. 'mad, bad, or possessed'? Perceptions of self-harm and mental illness in evangelical christian communities. Pastoral Psychology. 2022:No-Specified.
7. Lupton D. 'The Internet Both Reassures and Terrifies': exploring the more-than-human worlds of health information using the story completion method. Medical humanities. 2021;47(1):68-77.
8. Moller N, Tischner I. Young people's perceptions of fat counsellors: "How can THAT help me?". Qualitative Research in Psychology. 2019;16(1):34-53.
9. Nimbley E, Caes L, Jones A, Fisher E, Noel M, Jordan A. A linguistic analysis of future narratives in adolescents with Complex Regional Pain Syndrome and their pain-free peers. European journal of pain (London, England). 2021;25(3):693-703.
10. Olstein J, Finn MD. Daring to speak its name: Perceptions of suicidal ideation among australian gay men. Journal of Community Psychology. 2021:No-Specified.
11. Scholz B, Bocking J, Hedt P, Lu VN, Happell B. 'Not in the room, but the doctors were': an Australian story-completion study about consumer representation. Health promotion international. 2020;35(4):752-61.
12. Scott AG, Hunter SC, Johnson BJ. Exploring the social norms regarding parents' food provision in Australia using story completion methodology. Appetite. 2022;178:106165.
13. Shah-Beckley I, Clarke V. Exploring therapists' and psychology students' constructions of sexual refusal in heterosexual relationships: A qualitative story completion study. Counselling & Psychotherapy Research. 2021;21(4):946-56.
14. Tichenor CC, Rundall TG. Attitudes of physical therapists toward cancer: a pilot study. Physical therapy. 1977;57(2):160-5.
15. Tischner I. Tomorrow is the start of the rest of their life-So who cares about health? Exploring constructions of weight-loss motivations and health using story completion. Qualitative Research in Psychology. 2019;16(1):54-73.
16. Vaughan P, Lenette C, Boydell K. 'This bloody rona!': using the digital story completion method and thematic analysis to explore the mental health impacts of COVID-19 in Australia. BMJ open. 2022;12(1):e057393.
17. Walsh E, Malson H. Discursive constructions of eating disorders: A story completion task. Feminism & Psychology. 2010;20(4):529-37.
